# Effect of renal perfusion and structural heterogeneity on shear wave elastography of the kidney: an in vivo and ex vivo study

**DOI:** 10.1186/s12882-017-0679-2

**Published:** 2017-08-08

**Authors:** Xiaona Liu, Na Li, Tao Xu, Fang Sun, Rui Li, Qimin Gao, Lianxiang Chen, Chaoyang Wen

**Affiliations:** 1 Chinese PLA (People’s Liberation Army) Medical School, Beijing, 100853 People’s Republic of China; 2 Department of Ultrasound, Binzhou Medical University Hospital, Binzhou, Shandong 256603 People’s Republic of China; 3 Department of Auxiliary Diagnosis, The 463rd Hospital of Shenyang Military Region, Shenyang, Liaoning 110042 People’s Republic of China; 4 Department of Ultrasound, Chinese PLA Navy General Hospital, Beijing, 100048 People’s Republic of China; 5 Department of Ultrasound, Hainan Branch of Chinese PLA General Hospital, Sanya, Hainan 572013 People’s Republic of China

**Keywords:** Renal elasticity, Perfusion, Shear wave elastography, Young’s modulus, Anisotropy, Haemodynamics

## Abstract

**Background:**

To evaluate the effect of perfusion status on elasticity measurements of different compartments in the kidney using shear wave elastography (SWE) both in vivo and ex vivo.

**Methods:**

Thirty-two rabbit kidneys were used to observe the elasticity variation caused by renal artery stenosis and vein ligation in vivo, and six beagle kidneys were studied ex vivo to explore the effect of renal perfusion on elasticity. Supersonic SWE was applied to quantify the elasticity values of different renal compartments (cortex, medulla and sinus). Additionally, histopathological examination was performed to explore the possible mechanisms.

**Results:**

The elasticity of the cortex was higher than that of the medulla, and the elasticity of the sinus was lowest among the compartments in native kidneys. The Young’s modulus (YM) of the cortex, medulla and sinus increased gradually as the duration of renal vein ligation increased, from 16.34 ± 1.01 kPa to 55.06 ± 5.61 kPa, 13.71 ± 1.16 kPa to 39.63 ± 2.91 kPa, and 12.61 ± 0.84 kPa to 29.30 ± 2.04 kPa, respectively. In contrast, the YM of the three compartments respectively decreased with progressive artery stenosis, from 16.34 ± 1.83 kPa to 11.21 ± 1.79 kPa, 13.31 ± 1.67 kPa to 8.07 ± 1.37 kPa, and 12.78 ± 2.66 kPa to 6.72 ± 0.95 kPa. Artery perfusion was the main factor influencing elasticity in ex vivo. The cortical elasticity was more prone to change with renal perfusion both in vivo and ex vivo. Histopathological examination showed progressive changes in the structure and content of the three compartments, consistent with the elasticity variation.

**Conclusions:**

Both the complex structure/anisotropy and the perfusion of the kidney obviously influence the evaluation of renal elasticity. The measurement of SWE should be performed at a specific location along a certain angle or direction, and renal perfusion status should also be taken into account to ensure reproducible detection.

## Background

In the last decade, ultrasound shear wave elastography (SWE), an emerging non-invasive measurement method, has been successfully employed to aid the diagnosis of disease or to judge the stage of disease progression. The most frequent application of SWE has been in liver disease [1–3]. The technique has specifically been approved for discriminating between normal and cirrhotic liver tissue and is particularly valuable in assessing the severity of liver fibrosis [4].

Renal diseases, such as chronic kidney disease (CKD), and kidney transplantation are usually accompanied by interstitial fibrosis, and the degree of fibrosis is correlated with the severity of the disease. Several quantitative trials that used SWE to evaluate renal disease have yielded conflicting results [5–7]: the majority of prior studies suggested a positive correlation between SWE estimates and the presence of CKD or fibrosis [5, 6, 8], but others indicated that SWE does not reflect the stage of CKD [9] or rejection of renal transplantation [10–12].

Compared with the liver, the kidney has much higher tissue heterogeneity and anisotropy [10]. Kidney stiffness not only is related to fibrosis but also is sensitive to gender, age, the anatomical structure of the kidney, blood perfusion, body mass index (BMI), ureteral pressure, inflammation, and oedema, among other factors [13, 14]. With the exception of fibrosis, renal structure and perfusion, none of these factors has been identified as important in multiple studies, suggesting that the potential confounding effects are small or inconsistent. Certain studies have even indicated that the effects of blood flow and structural anisotropy exceed those of fibrosis [15–17]. Meanwhile, few studies have explored the relationship between renal structure/perfusion and elasticity. Warner et al. [17] found that the cortical stiffness detected by magnetic resonance elastography decreased with an acute decrease in renal blood flow (RBF). Similarly, in chronic renal arterial stenosis, a decrease in RBF offset a likely increase in stiffness secondary to the development of fibrosis. Gennisson et al. [18] found that intrarenal elasticity values varied with tissue anisotropy as well as with vascular and urinary pressure levels. However, the specific mechanism was not investigated, and the effects of an incremental decrease in arterial perfusion and of the degree of venous obstruction were not discussed.

In addition, due to the complexity of the location, structure and function of the kidney, a uniform standard for the appropriate elasticity range of normal kidneys is lacking [14]. As elasticity is an important factor influencing the diagnosis of renal disease via SWE, it is important to observe elasticity in different parts of the native kidney and to explore the possible mechanisms affecting renal elasticity.

Therefore, the purpose of the present study was to demonstrate the feasibility of using SWE to assess elasticity in different compartments of the kidney (cortex, medulla and sinus) and to explore the effect of structural complexity and differences in renal perfusion on elasticity measurements both in vivo and ex vivo.

## Methods

### Animal preparation

Sixteen healthy female Japanese white rabbits (ID: 11,400,800,001,403) with a weight of 3.0 ± 0.3 kg and 3 beagles (ID: 11,400,800,001,356) with a weight of 6.9 ± 0.5 kg were used following approval by the Institutional Care & Use Committee (IACUC) of The First Affiliated Hospital of Chinese PLA General Hospital [SYXK(JUN)2012–0014]. All animals were purchased from the laboratory animal department of The First Affiliated Hospital of Chinese PLA General Hospital (Beijing, China). The vendor provided health reports to certify that all rabbits were free of pathogens. All rabbits were individually housed in iron cages on a suspended floor, and the beagles were housed in a large iron cage on the floor under the same controlled conditions (temperature, 21 ± 2 °C; relative humidity, 50 ± 20%; air ventilation, 10 to 15 times per hour; artificial lighting, 12 h per day).

### Animal models

The 32 kidneys of the 16 rabbits were divided into 2 groups, as follows: group I, unilateral left renal vein occlusion in vivo (*n* = 8), and group II, unilateral left renal artery stenosis (RAS) in vivo (*n* = 8). And The contralateral right kidneys in group I (*n* = 8) and group II (*n* = 8) were used as respective controls of for the two groups, respectively. Group III contained six kidneys from 3 beagles for the ex vivo study (*n* = 6).

All animals were anaesthetized under 3 ml/kg 5% pentobarbital sodium (CAS:57–33-0, Merck, Germany), followed by midline laparotomy. In group I, the left renal vein was ligated for up to 90 min. In group II, the left renal artery was ligated after establishing a progressive reduction in RBF by 50% or 100% of baseline. In group III, both kidneys were removed immediately after sacrifice, and the renal arteries were perfused with normal saline containing 50 U/ml low-molecular-weight heparin calcium (Nadroparin Calcium, Hainan, China) to prevent blood coagulation. The kidney and the corresponding skin were coated with warm medical ultrasonic couplant in group I and group II. In group III, the kidney was directly coated with warm medical ultrasonic couplant.

### SWE imaging

**Fig. 1:**
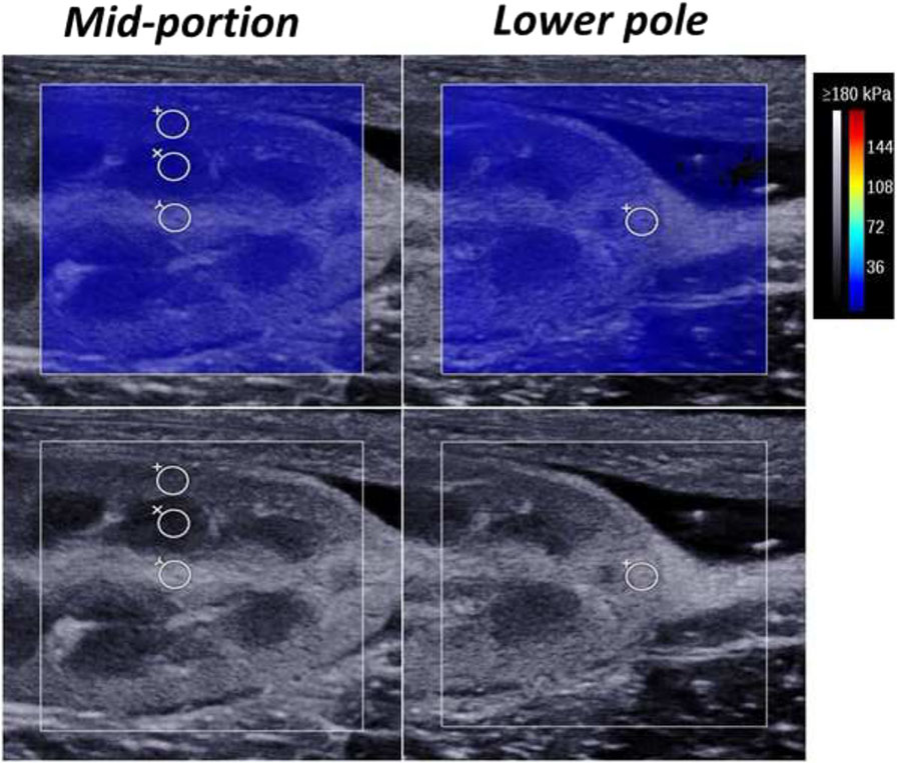
Representative images of elasticity measurement by shear wave elastography (SWE) in one kidney. The regions of interest (ROIs) were positioned in the cortex, medulla and sinus of the mid-portion and in the cortex of the lower pole using the B-mode image. The lower and upper rows show the changes in the ROI by grey-scale ultrasound and SWE, respectively. The colour map is the distribution of elasticity values scaled from 0 to 180 kPa and calculated from shear wave velocity (SWV) values using Young’s modulus (YM)

SWE was performed on both kidneys, and the mid-portion of each of the three compartments and the lower pole of the renal cortex were all measured when the ultrasound beam was placed along the main pyramid axis (Fig. 1).

All ultrasound examinations were performed with an Aixplorer ultrasound system (Supersonic Imagine, Aix-en-Provence, France) equipped with an SL 4–15 MHz transducer. The colour scale elasticity map had a 2-cm^2^ area and was positioned to cover an entire renal segment of interest. Elasticity values were quantified by using three 2-mm-diameter regions of interest (ROIs) within the cortex, medulla and sinus. Once the location of the target area was determined after optimizing the B-mode image, the Virtual Touch quantitative function was initiated. At each phase, one of the authors (Liu X. N.) repeated the measurement three times to calculate the mean value.

In group I, both sides of the kidneys were measured at 0 min, 15 min, 30 min, 60 min and 90 min after ligation of the left renal vein. In group II, the degree of artery stenosis was judged based on the ratio of peak systolic velocity between the stenosis and the proximal renal artery. Measurements were taken when artery stenosis reached 50% or 100% for 5 min. Renal length was determined as the maximum longitudinal dimension. In group III, regulative infusion elevations of 0 m, 1.0 m and 2.0 m were performed to provide different perfusion pressures (1 m is approximately equivalent to 75 mmHg). The ultrasound measurement was repeated when no ligation, ligation of the renal vein only, and combined ligation of the vein and ureter were performed, with a 5-min rest period and 15-min intervals.

### Histopathological examination

In group I, four left kidneys were randomly selected at 15 min, 30 min, 60 min and 90 min after ligation for histopathological examination. Similarly, in group II, two left kidneys were selected at 50% and 100% stenosis. Two contralateral kidneys in each of the two groups were considered as controls, respectively. For comparison with the SWE examinations, the mid-portion of each kidney was selected for the next procedure. In particular, all tissues were fixed in 10% neutral buffered formalin for 14 days, dried in alcohol solutions of increasing concentrations and finally embedded in paraffin. Using a microtome, slices with a thickness of 4–6 μm were then obtained and stained with haematoxylin and eosin (HE) according to a standard protocol. Histological analysis was performed by two experienced pathologists (with more than 5 years of experience in nephropathology) who were blinded to the clinical data and the SWE measurements.

### Statistical analysis

The results are expressed as the mean ± standard deviation (SD). Comparisons between group means were performed using a paired Student’s *t*-test, one-way analysis of variance (ANOVA) or repeated measures. Bonferroni correction was used for multiple comparisons. The statistical significance level was defined as two-sided *P* < 0.05. All statistical analyses were performed with SPSS statistical software, version 19.0 (IBM Corp., Armonk, NY, USA).

## Results

### Elasticity measurement in normal kidneys

Considering the complexity of the kidney architecture, the corresponding Young’s modulus (YM) was measured in different compartments (cortex, medulla and sinus) in both kidneys of all rabbits (Table 1). The mid-portion of each of the three compartments and the lower pole of the renal cortex were all measured when the ultrasound beam was placed along the main pyramid axis. The mean YM values of the cortex, medulla and sinus were respectively 16.33 ± 1.44 kPa, 13.55 ± 1.38 kPa, and 12.23 ± 1.42 kPa in the mid-portion of the right kidney and 15.78 ± 1.52 kPa, 13.16 ± 1.69 kPa, and 11.70 ± 1.62 kPa in the left kidney. One-way ANOVA indicated significant differences among the intrarenal compartments (all *P* < 0.05), whereas there was no significant difference in any compartment between the left and right kidneys (all *P* > 0.05). The mean YM of the lower pole was 4.50 ± 0.48 kPa in the right kidney and 4.41 ± 0.36 kPa in the left kidney (*P* = 0.540). The elasticity values of the lower pole were significantly lower than those of the mid-portion (both *P* < 0.001).

**Table 1:** Measurement of normal renal elasticity by shear wave elastography (SWE)

Position	Compartments	Left kidney (kPa, *n* = 16)	Right kidney (kPa, *n* = 16)	*P* value
Renal mid-portion	Cortex	15.78 ± 1.52^**##^	16.33 ± 1.44^**##^	0.382
	Medulla	13.16 ± 1.69^*^	13.55 ± 1.38^*^	0.561
	Sinus	11.70 ± 1.62	12.23 ± 1.42	0.432
Renal lower pole	Cortex	4.41 ± 0.36	4.50 ± 0.48	0.54

Values are given as the mean ± SD. Measurements were performed respectively with the ultrasound (US) beam along (axial, mid-portion) and perpendicular (transverse, lower pole) to the main axis of the pyramid. Using paired *t*-test or ANOVA analysis. Compared with sinus: ^*^, *P*<0.05; ^**^, *P*<0.01 Compared with medulla: ^##^, *P*<0.01

### Elasticity variations caused by ligation of the renal vein in vivo

**Fig. 2:**
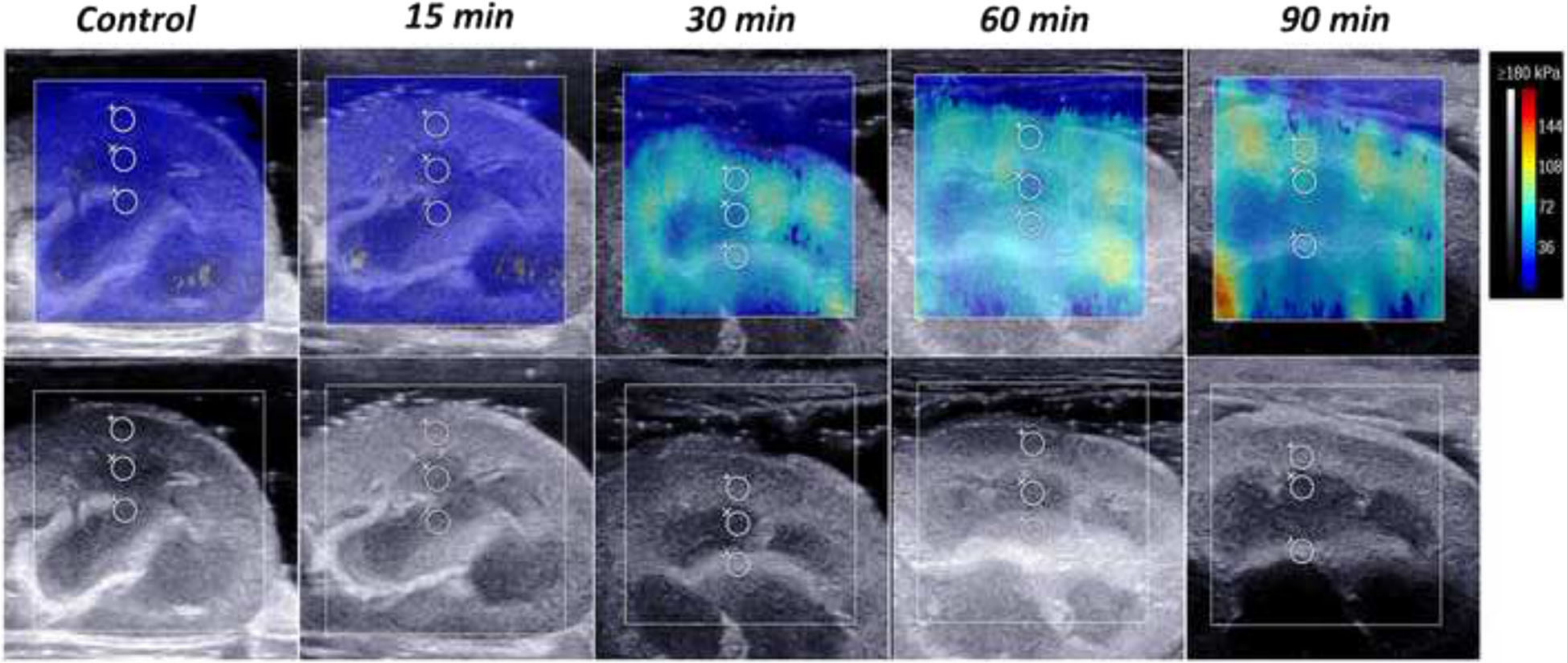
Representative images showing the progressive increase in stiffness by SWE in one kidney when the renal vein was occluded. The lower and upper rows show the changes in the ROI by grey-scale ultrasound and SWE, respectively. The colour map is the distribution of elasticity values scaled from 0 to 180 kPa

The stiffness of the three compartments increased gradually with the extension of the duration of ligation of the renal vein (Fig. 2). The mean length of the kidneys varied from 6.93 ± 1.19 cm to 10.05 ± 0.16 cm, and the YM values of cortex, medulla and sinus varied from 16.34 ± 1.01 kPa to 55.06 ± 5.61 kPa, 13.71 ± 1.16 kPa to 39.63 ± 2.91 kPa, and 12.61 ± 0.84 kPa to 29.30 ± 2.04 kPa, respectively (Fig. 4a, c). The elasticity of the cortex and medulla increased significantly from 15 min (*P* < 0.01), and the increase was most obvious at 60 min (Table 2). The elasticity of the sinus increased significantly after 30 min (*P* = 0.003) and remained stable thereafter. The increase in the elasticity of the cortex was significantly higher than that observed for the medulla (*P* = 0.030) or sinus (*P* = 0.031) in the first 30 min, whereas no significant difference was observed between the medulla and sinus (*P* = 1.000). The increase in the elasticity of the cortex was also larger than sinus (*P* = 0.001) after 30 min, while the difference with medulla was not significant (*P* = 0.065). Finally, the renal length increased significantly in the first 30 min (6.93 ± 1.19 cm to 10.34 ± 1.53 cm, *P* < 0.001) and remained stable thereafter. No changes were observed in the three compartments of the contralateral kidney throughout the experiment.

**Table 2:** Measurement of YM in the renal cortex, medulla and sinus of kidneys with ligation of the renal vein or renal artery in vivo

Positions	Vein ligation (*n* = 8)					Artery ligation (*n* = 8)		
	No ligation	15 min	30 min	60 min	90 min	No ligation	Stenosis	Occulation
Cortex (Kpa)	16.34 ± 1.01	24.28 ± 1.34^**^	34.73 ± 2.36^**^	47.52 ± 4.67^**^	55.06 ± 5.61^**^	16.34 ± 1.83	14.26 ± 2.48	11.21 ± 1.79^**^
Medulla (Kpa)	13.71 ± 1.16	17.37 ± 1.43^**^	25.39 ± 2.86^**^	34.57 ± 6.64^**^	39.63 ± 2.91^**^	13.31 ± 1.67	11.32 ± 2.97	8.07 ± 1.37^**^
Sinus (Kpa)	12.61 ± 0.84	16.14 ± 4.04	24.20 ± 5.96^**^	26.87 ± 7.00^**^	29.30 ± 2.04^**^	12.78 ± 2.66	10.39 ± 2.57^*^	6.72 ± 0.95^**^

Values are given as the mean ± SD. Measurements were obtained at the mid-portion of the kidneys. Using paired *t*-test, compared with pre-ligation. ^*^ *P* < 0.05, ^**^ *P* < 0.01

### Effects of RAS on elasticity in vivo

**Fig. 3:**
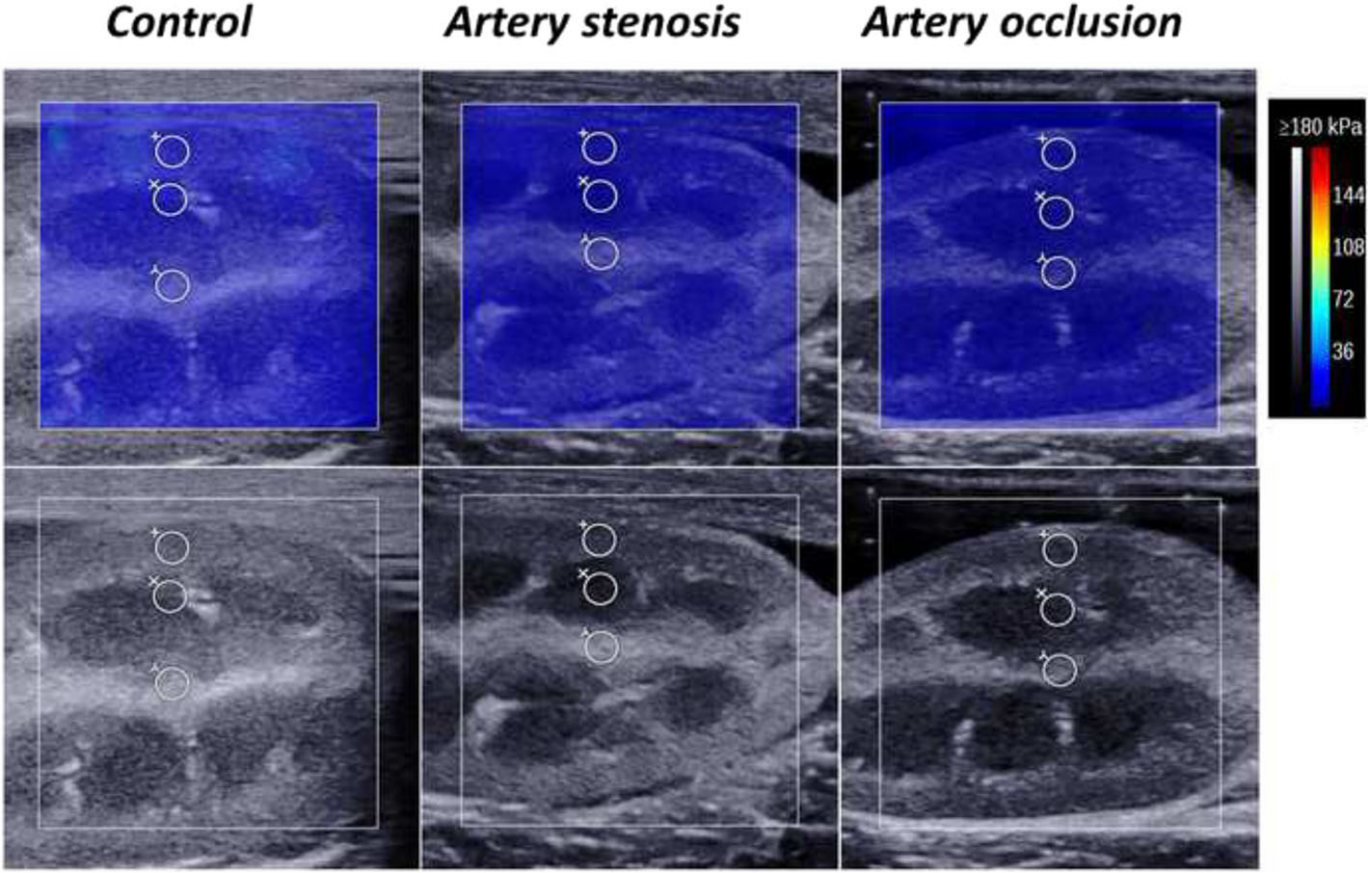
Representative images showing the progressive decrease in stiffness by SWE in one kidney with different degrees of stenosis of the renal artery. The lower and upper rows show the changes in ROI by grey-scale ultrasound and SWE, respectively. The colour map is the distribution of elasticity values scaled from 0 to 180 kPa

**Fig. 4:**
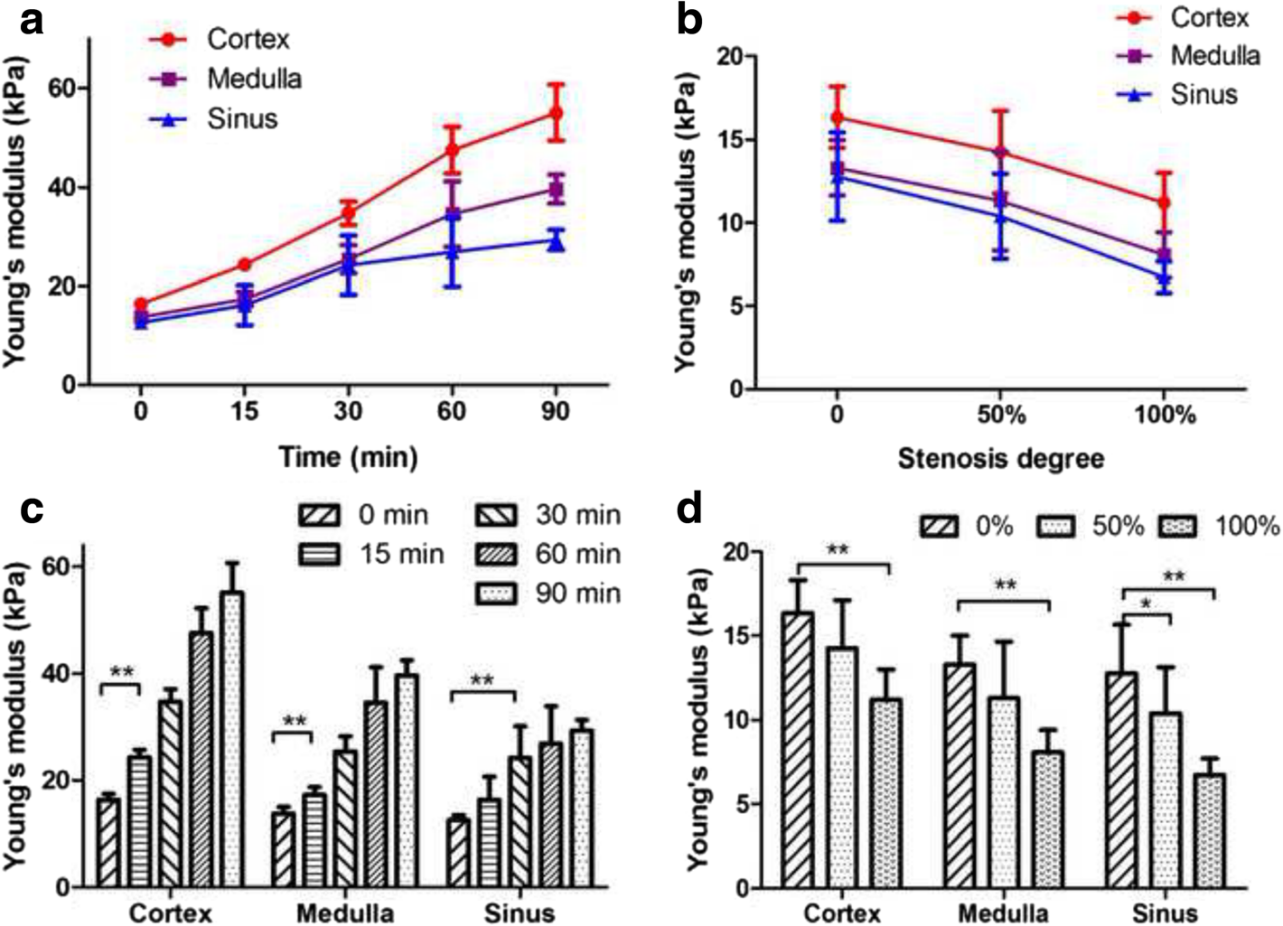
Trends of elasticity variation of the renal cortex, medulla and sinus caused by ligation of the renal vein and renal artery in vivo. The curves and histograms show the variation of YM in each compartment after ligation of the renal vein (**a**, **c**) and renal artery (**b**, **d**), respectively. Data are expressed as the mean ± SD. Intergroup comparisons were performed by paired *t*-test. *P < 0.05, **P < 0.01

The elasticity of the three compartments decreased gradually with increasing RAS (Fig. 3). Figure 4b shows the variation trends of the renal length and mean YM of 8 left kidneys with different degrees of stenosis (0%, 50% and 100%). The kidney length gradually decreased (7.61 ± 0.97 cm to 6.54 ± 0.90 cm, *P* = 0.023), and the YM of the cortex, medulla and sinus decreased from 16.34 ± 1.83 kPa to 11.21 ± 1.79 kPa, 13.31 ± 1.67 kPa to 8.07 ± 1.37 kPa, and 12.78 ± 2.66 kPa to 6.72 ± 0.95 kPa, respectively (Table 2). The elasticity of all three compartments decreased after 50% RAS, and significant variation occurred after complete occlusion (*P* = 0.004, *P* = 0.001, and *P* = 0.003, respectively). There was no significant difference in the degree of decline among the three compartments (*P* = 1.000). The elasticity of the contralateral kidneys remained unchanged during RAS.

### Elasticity variation with varying renal perfusion ex vivo

**Fig. 5:**
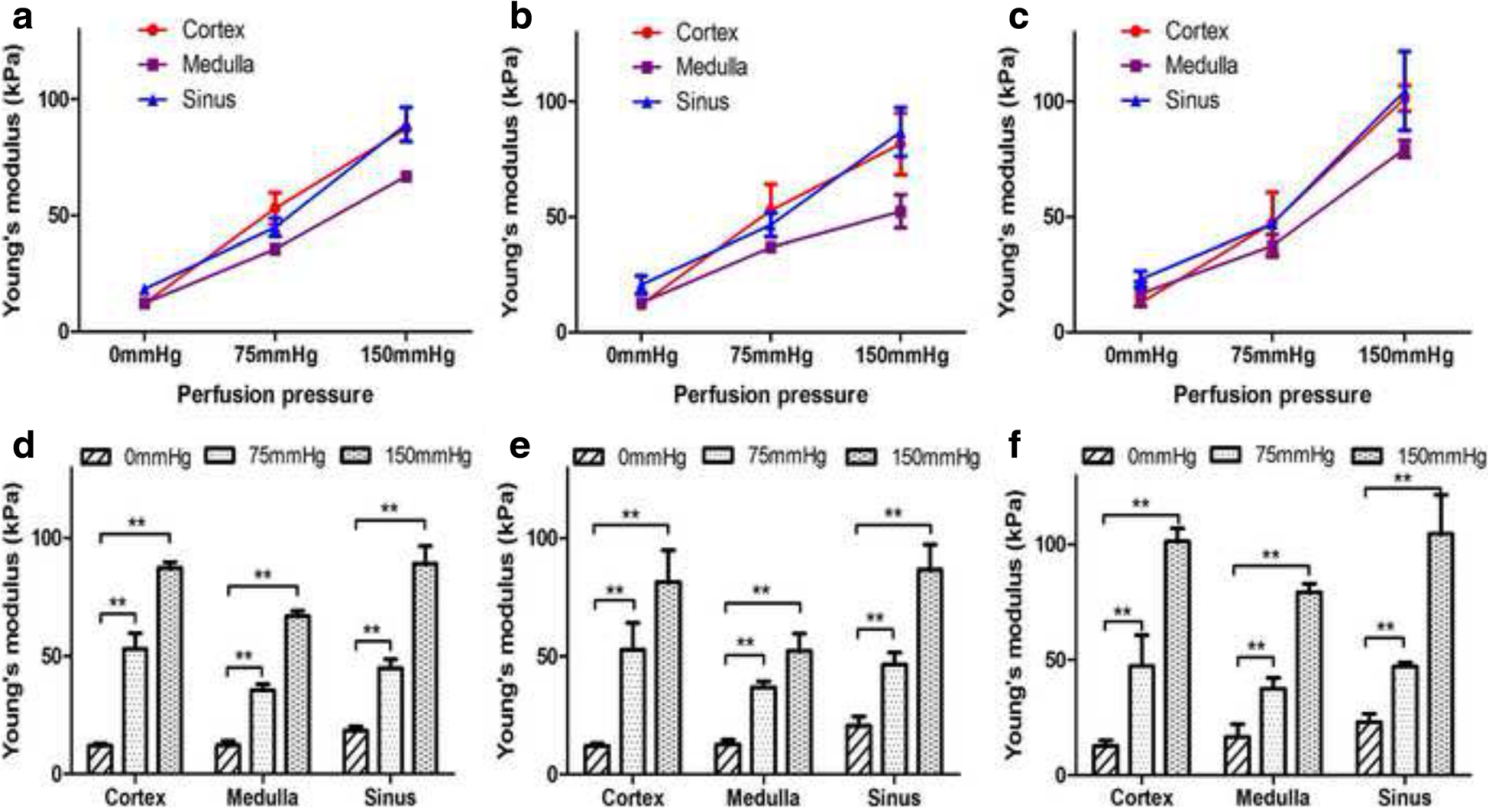
Trends of elasticity variation of the renal cortex, medulla and sinus with different perfusion pressures of the renal artery and ligation status in ex vivo. The curves and histograms show the variation of YM in each compartment when different perfusion pressures were applied under no ligation (**a**, **d**), ligation of the renal vein only (**b**, **e**) and ligation of both the renal vein and ureter (**c**, **f**), respectively. Data are expressed as the mean ± SD. Intergroup comparisons were performed by paired *t*-test. **P < 0.01

The YM values of the three compartments of the kidney were measured with varying renal perfusion in six beagles ex vivo (Fig. 5). Regardless of the ligation state (no ligation, renal vein only and combined ligation with ureter), the YM values of the renal cortex, medulla and sinus all increased significantly with the elevating artery perfusion from 0 mmHg to 150 mmHg (Table 3). When 75 mmHg was firstly applied, the increase of YM in the renal cortex was larger than the increased in the medulla and sinus, and the difference was statistically significant at no ligation and vein ligation (*P* all <0.01). The increase in cortex was also larger than those of the medulla and sinus when combined ligation with ureter, although the difference was relatively small (*P* = 0.027 and *P* = 0.108, respectively). However, the increase of sinus became more obviously compared with the cortex and medulla when the perfusion pressure was elevated to 150 mmHg at no ligation (*P* = 0.037 and *P* = 0.006) and vein ligation (*P* = 0.408 and *P* = 0.012). There was no difference of elastic increase among three compartments when the renal vein and ureter were simultaneously ligated (*P* = 0.141).

**Table 3:** Measurement of YM in the renal cortex, medulla and sinus of beagle’s kidneys with varying renal perfusion ex vivo

Ligation status	Cortex (Kpa, *n* = 6)			Medulla (Kpa, *n* = 6)			Sinus (Kpa, *n* = 6)		
	0 mmHg	75 mmHg	150 mmHg	0 mmHg	75 mmHg	150 mmHg	0 mmHg	75 mmHg	150 mmHg
No ligation	12.23 ± 0.83	52.99 ± 6.77^**^	87.50 ± 2.36^**^	12.26 ± 1.88	35.56 ± 2.48^**^	66.95 ± 2.34^**^	18.32 ± 1.91	44.86 ± 3.86^**^	89.23 ± 7.39^**^
Vein ligation	12.15 ± 1.06	52.79 ± 11.37^**^	81.63 ± 13.33^**^	12.81 ± 1.91	36.85 ± 2.48^**^	52.39 ± 7.20^**^	20.65 ± 3.91	46.48 ± 5.19^**^	86.81 ± 10.48^**^
Combined ligation	12.79 ± 2.48	47.31 ± 13.40^**^	101.39 ± 5.61^**##^	16.70 ± 5.36	37.59 ± 4.72^**^	79.42 ± 3.68^**##^	23.06 ± 3.59	47.12 ± 1.78^**^	104.60 ± 17.00^**^

Values are given as the mean ± SD. Measurements were obtained at the mid-portion of the kidneys. Using paired *t*-test or ANOVA analysis. Compared with 0 mmHg: ^**^ *P*<0.01. Compared with no ligation: ^##^ *P*<0.01

We also compared the effects of different ligation statuses (no ligation, renal vein only and combined ligation with ureter) under the same perfusion pressure on the elasticity of the cortex, medulla and sinus. When the perfusion pressure was maintained at 75 mmHg, there were no difference of elasticity in the cortex, medulla and sinus among three ligation statuses (*P* = 0.601, *P* = 0.589 and *P* = 0.593, respectively). After increasing the perfusion pressure to 150 mmHg, compared with no ligation, the YM of the cortex and medulla increased dramatically only when the renal vein and ureter were ligated simultaneously (*P* = 0.001 and *P* = 0.001). The YM of sinus also increased obviously when combined ligation with ureter, although the difference was not significant (*P* = 0.141).

### Histopathological changes

**Fig. 6:**
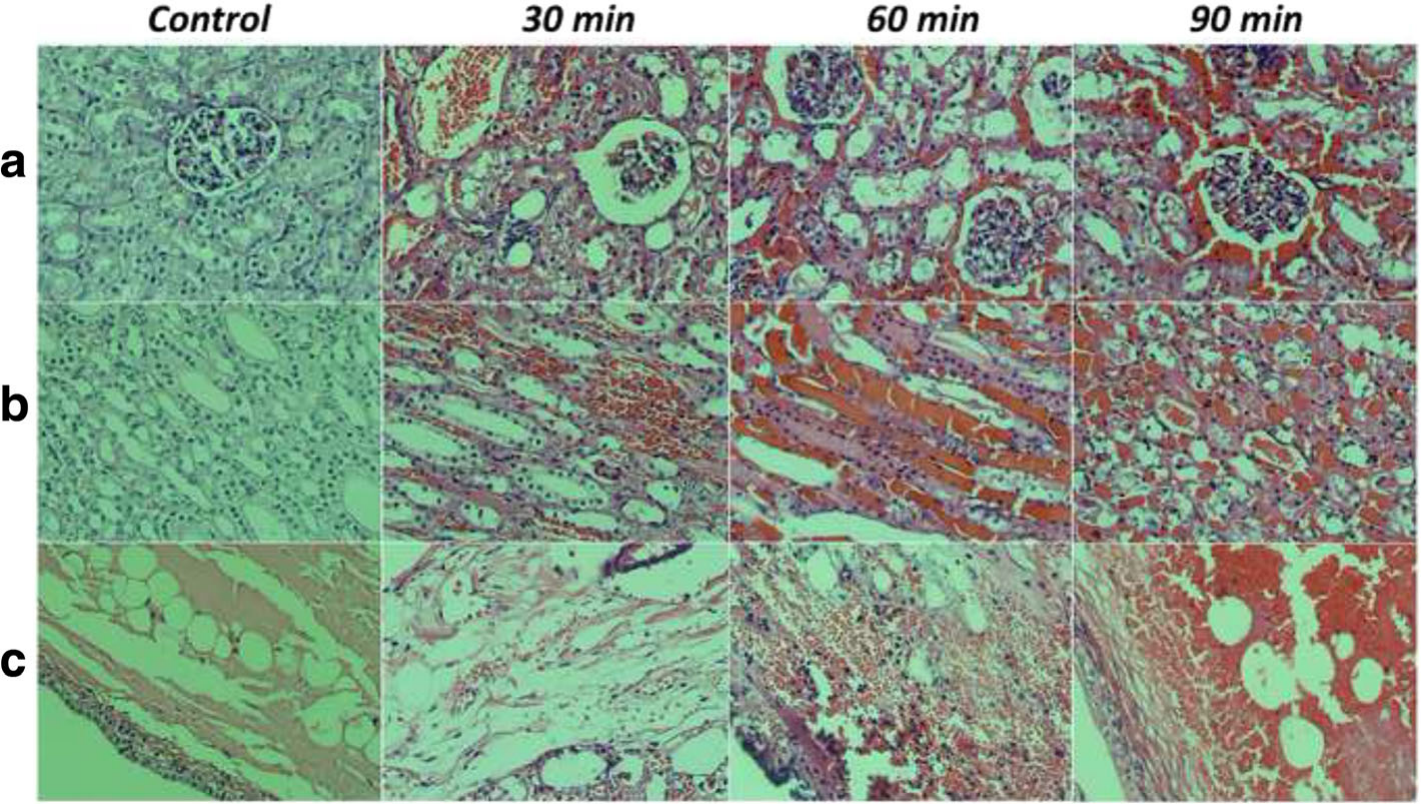
Representative histopathological sections of the renal cortex, medulla and sinus in kidneys of the control and 30 min, 60 min, and 90 min after ligation of the renal vein in vivo. Compared with the control, the structure of the renal cortex (**a**), medulla (**b**) and sinus (**c**) exhibited progressive changes with increasing congestion time. Haematoxylin and eosin (HE) staining was performed (original magnification, ×400)

As the duration of vein ligation increased, the kidney volume and film tension gradually increased, and the colour deepened. The histopathological features are shown in Fig. 6. In the first 30 min after ligation, the boundaries of the cortex and medulla remained clear, and the glomerular volume increased with aggregation of red blood cells (RBCs). Renal tubular epithelial cells exhibited hydropic and granular degeneration, and congestion was observed in the renal interstitium. The sinus also showed mild haemorrhage and congestion. At 60 min, obvious changes occurred. The morphology of the glomerulus and tubule grew vague, with pink protein-like substances deposited in the capsule. A large amount of protein and haemoglobin blocked the tubular lumen, which was more obvious in the medulla, with abundant erythrocyte accumulation. The degree of interstitial haemorrhage and vascular congestion was significantly aggravated, and the sinus was also affected. At 90 min, glomerular collapse was observed, and the tubular epithelial cells presented diffuse necrosis and disintegration. Obvious subcapsular haematoma, severe interstitial haemorrhage and vascular congestion were also observed. Additionally, the renal sinus was filled with RBCs, and thrombosis was observed.

As RAS increased, the kidney gradually shrunk, the film tension decreased, and the colour lightened. Microscopic observation (data not shown) revealed no significant alteration of renal structure at 50% stenosis, except for the reduced blood volume. When stenosis reached 100%, the kidney volume decreased, and fewer RBCs were present. The tubular epithelial cells showed mild hydropic degeneration, and the structure became obscure. Moreover, interstitial blood flow was reduced significantly.

## Discussion

Compared with conventional ultrasound elastography, SWE possesses particular advantages, including real-time acquisition of shear wave propagation and multiple ROIs with variable shapes, allowing its use for the accurate localization and evaluation of kidney elasticity. However, multiple studies have suggested that quantification using SWE is more complex and variable within the kidney than the liver because of the kidney’s higher structural anisotropy and blood perfusion. Kidney stiffness is related to fibrosis as well as mechanical and functional parameters such as anisotropy, vascularization, and hydronephrosis [19–21]. In addition, variations between different operators, pathophysiological processes and detection methods can influence the evaluation of elasticity. There is also no consensus on reference values for the native kidney [14].

In the present study, the elasticity/YM of the three compartments showed good agreement between the two sides of the kidney. However, considerable intrarenal differences between different compartments were observed. In particular, the elasticity of the mid-portion of the renal cortex was significantly higher than that of the lower pole. This difference might be related to the angle between the sound beam and the cortex, i.e., anisotropy [21]. Bob F et al. reported that the success rate of measurements in the renal poles was low compared with measurements in the mid-portion of the renal parenchyma and suggested that the renal poles should be avoided during measurements to ensure good reproducibility [22]. In the current study, the elasticity of the renal cortex in the mid-portion was obviously greater than that observed for the medulla, and the elasticity of the renal sinus was lowest, consistent with previous studies [7, 18, 19]. These differences might be due to the specific components of the compartments [23]: the renal cortex consists of proximal and distal tubules and renal glomeruli; the medulla mostly consists of the loop of Henle and the lower part of the collecting tubule; and the renal sinus is composed of the renal calyx, blood vessels, nerves and loose connective tissue [16]. These architectural discrepancies result in multi-axial characteristics and different elasticity values. Previous studies have found that the architecture of the parenchyma is highly oriented and that the elasticity values differ when ultrasound beams are parallel or perpendicular [18].

RBF is another influencing factor. The kidney is a richly perfused organ, with an eighth of the cardiac blood flow distributed into each kidney, and the degree of vascular pressure has an influence on elasticity values [16]. The cortex in particular is highly vascularized, with 94% of all RBF, and renal vascular resistance resides mainly in the cortical microvessels [17]. By contrast, the proportion of RBF in the medulla is much lower, namely, only 6%. Considering the complexity of the renal structure, SWE detection of the kidney should be aimed at a specific location and along a specific direction/angle to obtain reliable and reproducible results [14].

In the current study, after ligation of the renal vein, the elasticity of the three compartments increased with time, which might be related to the following factors. ①The blocked vein reflux resulted in blood stasis in the intra-renal vein and peritubular capillary, which increased the intravascular hydrostatic pressure. The precipitation of visible components in the renal tubules also increased. The sustained pipeline congestion reduced the tissue deformation force and accordingly increased tissue hardness. All of the above factors contributed to the increase in renal strain, and these changes might be more obvious in the parenchyma because of its high compactness. ②Hypoxia-ischaemia caused by congestion resulted in tissue oedema in the renal tubular system and interstitium, which also increased the stiffness of the kidney [24]. ③The formation of microthrombi in the renal vein might also have participated in increasing kidney stiffness. When the kidney was removed, we observed small blood clots in the renal vein. However, because pathology cannot distinguish thrombi from congestion blots, further study is required. ④The elasticity increase in the cortex was much higher than the increases in the medulla and sinus, consistent with a previous report by Gennisson JL [18]. One explanation might be the different degrees of vascular pressure [19]. As noted above, the kidney is highly vascularized, mainly in the cortex, so the cortex might be predominantly affected when the renal perfusion changes. Meanwhile, the stiffness of the medulla and sinus might be far less prone to changing with renal perfusion pressure because of their lower RBF at baseline [18].

With increasing RAS, the YM of the cortex, medulla and sinus decreased nearly linearly, and significant variation occurred after complete occlusion. This variation tendency is highly consistent with a previous report [17]. Meanwhile, the pathological results in the present study revealed no significant changes in renal structure except for the obviously decreased RBF. It might be speculated that the decreased elasticity was mainly related to the reduction of RBF because the duration of stenosis was too short to cause structural changes. As reported previously, up to 25% of renal volume may be attributable to blood pressure and blood, filtrate and urine content, implying a substantial contribution of haemodynamics to renal turgor [25]. The extent of the decrease in elasticity remained consistent among the three compartments, possibly due to the great differences in the structural composition of different compartments. Although the renal cortex receives more than 90% of RBF, the blood is dispersed throughout the vast capillary network. The RBF to the medulla and renal sinus is lower than that to the cortex but is concentrated in far fewer blood vessels. The strain variation caused by the RBF to the cortex might be similar to what occurs in the medulla and renal sinus. Decreased RBF also activates intrarenal regulatory mechanisms, which regulate the blood flow and intravascular pressure; this might in turn affect renal strain and elasticity.

Considering the complex regulatory mechanisms of body fluids and the endocrine system in vivo, we further established an ex vivo model to explore the effect of renal perfusion on kidney elasticity. The elasticities of the three compartments all increased in a linear manner with elevated artery perfusion pressure, in good agreement with the variation trends after RAS in vivo. Compared with the changes in elasticity caused by artery perfusion, ligation of the renal vein or combined ligation of the vein and ureter only induced mild variation of elasticity in each compartment, which indicated that the artery perfusion pressure was still the major factor influencing renal elasticity. Further, when a lower perfusion (75 mmHg) was applied, there was no significant difference of YM among three ligation statuses. When the perfusion pressure was elevated to 150 mmHg, vein ligation alone seemed to have no influence on elasticity, the YM of each compartment increased obviously only after the combined ligation of vein and ureter. It suggests that in addition to perfusion pressure, hydronephrosis is an important factor influencing renal elasticity [18]. The influence of vein ligation on renal elasticity was much smaller ex vivo than in vivo. One possible explanation is that the in vivo regulatory mechanism leads to intrarenal blood redistribution, which in turn causes changes in renal components and structure and influences renal strain and elasticity, as mentioned above. However, this effect was lost ex vivo, and the ligation of the renal vein might only block reflux and increase renal filtration. Further study is needed to obtain a more tangible explanation.

In the present study, higher perfusion pressure (150 mmHg) in the ex vivo model induced greater elasticity variation compared with what was observed in vivo*.* Although blood pressure fluctuates with the cardiac cycle in vivo, renal arterioles show a good pressure-protective effect, which may relieve the effect of perfusion pressure fluctuations on renal microcirculation. However, this protective effect cannot function ex vivo. In the current study, the external perfusion pressure was directly applied to the kidney microcirculation, which increased the internal tension of the tissue and the corresponding YM.

There are certain limitations to our study. First, the in vivo experiment explored the variation of renal elasticity over a limited time period and thus did not completely reflect actual changes in clinical practice with a longer disease course and more complicated pathophysiological processes. Second, normal saline was used for artery perfusion in the ex vivo experiment. The colloid osmotic pressure of saline differs greatly from renal perfusion in vivo, and hypoxia-ischaemia also induces the impairment of renal structure and function, which might underlie the discrepancy in the elasticity evaluation. Third, interobserver variability was not assessed, as all SWE examinations were performed by a single radiologist. Furthermore, this was a small-sample study, and the ex vivo experiment lacked pathological control tissues. In addition, given that our hypothesis was based on haemodynamic changes in the kidney, parameters directly related to RBF and renal vessel resistance should be evaluated in the future.

## Conclusions

The present study is the first to systematically explore the effects of increased renal perfusion and different tissue compartments on the evaluation of kidney elasticity by SWE both in vivo and ex vivo by analysing both mechanistic factors and pathological organization. Our study revealed obvious differences/anisotropy among the different renal compartments and that the YM value of each compartment was significantly affected by renal haemodynamics. The mechanism of elasticity variation might involve changes in strain related to the perfusion pressure, renal structure and kidney contents. Our study thus sheds further light on the impact of multiple confounding factors on the measurement of kidney elasticity, which will be of great importance to guiding the clinical application of SWE in the diagnosis and dynamic monitoring of disease.

## Abbreviations

ANOVA: Analysis of variance
CKD: Chronic kidney diseases
HE: Hematoxylin eosin
RAS: Renal artery stenosis
RBC: Red blood cell
RBF: Renal blood flow
ROI: Region-of-interest
SD: Standard deviations
SWE: Shear wave elastography
YM: Young’s modulus
